# Mesh Denoising via Adaptive Consistent Neighborhood

**DOI:** 10.3390/s21020412

**Published:** 2021-01-08

**Authors:** Mingqiang Guo, Zhenzhen Song, Chengde Han, Saishang Zhong, Ruina Lv, Zheng Liu

**Affiliations:** 1 School of Geography and Information Engineering, China University of Geosciences, Wuhan 430074, China; guomingqiang@mapgis.com (M.G.); songzhenzhen.sky@mapgis.com (Z.S.); hanchengde@cug.edu.cn (C.H.); ruinalv@cug.edu.cn (R.L.); 2 Key Laboratory of Urban Land Resources Monitoring and Simulation, Ministry of Natural Resources, Shenzhen 518000, China; 3 National Engineering Research Center of Geographic Information System, China University of Geosciences, Wuhan 430074, China; 4 School of Earth Resources, State Key Laboratory of Geological Processes and Mineral Resources, China University of Geosciences, Wuhan 430074, China; saishang@cug.edu.cn; 5 State Key Laboratory of Geological Processes and Mineral Resources, China University of Geosciences, Wuhan 430074, China

**Keywords:** mesh denoising, guided normal filtering, feature preserving, graph-cut, bilateral filtering

## Abstract

In this paper, we propose a novel guided normal filtering followed by vertex updating for mesh denoising. We introduce a two-stage scheme to construct adaptive consistent neighborhoods for guided normal filtering. In the first stage, we newly design a consistency measurement to select a coarse consistent neighborhood for each face in a patch-shift manner. In this step, the selected consistent neighborhoods may still contain some features. Then, a graph-cut based scheme is iteratively performed for constructing different adaptive neighborhoods to match the corresponding local shapes of the mesh. The constructed local neighborhoods in this step, known as the adaptive consistent neighborhoods, can avoid containing any geometric features. By using the constructed adaptive consistent neighborhoods, we compute a more accurate guide normal field to match the underlying surface, which will improve the results of the guide normal filtering. With the help of the adaptive consistent neighborhoods, our guided normal filtering can preserve geometric features well, and is robust against complex shapes of surfaces. Intensive experiments on various meshes show the superiority of our method visually and quantitatively.

## 1. Introduction

A triangulated mesh is one of the typical data types for representing 3D models. Commonly, triangulated meshes can be generated by using the original 3D coordinate datas that collected by 3D model scanning equipments, such as Kinect, laser scanner, CT, etc. However, there are many noises in the original 3D coordinate datas, and the noises also are generated in the 3D models reconstruction process [1,2]. These noises will bring challenges to 3D models visualization, splitting, spatial analysis, object extraction and 3D printing etc. [3,4,5]. Therefore, it is very significant to eliminate noises in triangulated meshes. The key issue is how to retain the original geometric structures and fine details when eliminate the noises. This problem becomes more challenging for the surfaces including complex shapes (e.g., narrow structures, multi-scale features, and fine details).

Over recent decades, the filtering methods have been widely used in mesh denoising, and can be roughly divided into isotropic and anisotropic filtering methods. The classical isotropic methods [6,7] mainly focus on removing the surface noise, but they neglect to preserve geometric features during the filtering process. Thus, these isotropic methods tend to produce denoised results with significant shape distortion. To address this issue, many anisotropic filtering methods [8,9,10,11,12,13,14,15,16,17,18,19,20] have been proposed. Bilateral filtering is a representative method in these anisotropic methods, which has been successfully applied in image processing for its ability of preserving features. Due to the success of bilateral filtering in image processing, it has been extended to geometry processing. Fleishman et al. [12] proposed a bilateral mesh denoising, which can directly remove noise via smoothing vertex positions. Zheng et al. [15] proposed a bilateral normal filtering (BNLF) by using normal filtering followed by vertex updating. Although the bilateral normal filtering proposed in [15] can preserve geometric features to some extent, it cannot effectively preserve sharp features, multi-scale features, and fine details in the case of high noise. The reason may be as follows. The bilateral normal filtering lacks a reliable guidance normal field to facilitate the filtering process. Thus, Zhang et al. [21] presented a patch-shift method to compute the guidance normal field for facilitating the bilateral normal filtering. Their method can preserve sharp features well, but may blur small-scale features and fine details because of their consistent neighborhoods constructing strategy. More specifically, when the surface contains complex shapes (e.g., narrow structures, multi-scale features, fine details), the uniformly constructed neighborhoods by their method inevitably contain geometric features in them, which further causes these contained features to be blurred in the normal filtering process. Thus, it is still an open problem to find an effective strategy to construct local neighborhoods that avoid including any geometric features. With the help of the well constructed local neighborhoods (without any geometric features contained), we can compute a guide normal field that strictly matches the underlying shape of the surface, which will greatly improve the results of the guided normal filtering.

In recent years, optimization-based methods are another kind of technique for mesh and image denoising. In order to preserve sharp features, the sparse optimization methods have been widely applied [3,5,22,23,24,25,26,27,28]. He and Schaefer [22] extended $L_{0}$ minimization to triangulated meshes for recovering piecewise constant surfaces. Zhang et al. [23] and Wu et al. [24] applied TV (total variation) regularization to mesh denoising for its edge-preserving property. Although the above $L_{0}$ and $L_{1}$ minimization methods achieve impressive results for preserving sharp features, they inevitably suffer from the undesire staircase artifacts in smoothly curved regions. In particular, this drawback is more severe for $L_{0}$ minimization [22,29], which may flatten some weak features and produce false edges in smoothly curved regions. Liu et al. [25] and Zhong et al. [26,30] proposed high-order based methods to overcome the above limitations of works [22,23,24,29]. Their methods can preserve sharp features and simultaneously recover smoothly curved regions well. However, in the presence of high noise, these high-order based methods may smooth sharp features and blur fine details. Many low-rank optimization methods [31,32,33,34] were introduced in mesh denoising to recover pattern similarity patches of the underlying surface. Unfortunately, these low-rank based methods cannot preserve sharp features well. In addition, because of the multi-patch collaborative mechanism, these low-rank based methods may be computationally intensive sometimes.

More recently, learning-based methods [35,36,37,38] have been gaining widespread attention, which have an advantage of parameters adjustment-free. Wang et al. [35] proposed a cascade normal regression (CNLR) method. The relation between the filtered results and the ground-truth was learned by CNLR. The advantage of this novel method is that there was no need to adjust parameters manually to eliminate noises. Generally, the performance of this method is well for small-scale noises, but it is not capable to dispose large-scale noises. In order to retain the geometrical structure features and fine details of the textures, Wang et al. [36] put forward a two-stage leaning method. Firstly, the face normal relation between the models with noises and the ground-truth was learned and the noises were eliminated by machine leaning. Secondly, the geometrical structure features and fine details of the textures were recovered by machine learning, so as to solve the object blurring issue generated in the first stage. Although these learning-based methods are free of parameter-tuning, and preserve geometric features well, they are highly dependent on the completeness of the training data set.

As we have seen, it is still quite challenging to preserve geometric features while removing noise, especially when the noisy mesh containing complex shapes (e.g., narrow structures, multi-scale features, and fine details). In view of these issues, we propose a guided normal filtering based on adaptive consistent neighborhoods for mesh denoising. The adaptive consistent neighborhoods are constructed by a proposed two-stage scheme. In the first stage, we design a consistency measurement to select the local neighborhood of each face (called the coarse consistent neighborhood) with the most consistent normal orientations. Then, a graph-cut based approach is iteratively performed to construct the final consistent neighborhood without any features contained. By using the constructed adaptive consistency neighborhoods, we can easily get the guidance normal field of the surface for restoring the noisy normal field. Following the guided normal filtering, we reconstruct vertex positions to match the filtered normal field. Taking a noisy mesh as input, our mesh denoising can recover complex shapes of the surface well while removing noise. Specifically, the main contributions of this paper are listed as follows:

- A reliable consistency measurement is designed to explicitly select the coarse consistent neighborhood containing the fewest features, thus providing a favorable neighborhood for each mesh face toward features-preserving effect. Then, a graph-cut based scheme is proposed, which can adaptively construct the more accurate neighborhood that does not contain any features. We can use the constructed consistent neighborhoods to compute a more accurate guide normal field.
- A guided normal filtering method via the adaptive consistent neighborhoods is proposed to restore the noisy normal field. We show the performance of our method on synthetic data including CAD and non-CAD meshes and a variety of scanned data acquired by the laser scanners and Kinect sensors. Experiments demonstrate that our method outperforms the existing state-of-the-art mesh denoising methods qualitatively and quantitatively.

The rest of the paper is organized as follows. In Section 2, we detail our guided normal filtering method based on constructing adaptive consistent neighborhoods. Then, our visual and numerical results are given in Section 3, and we discuss our mesh denoising method in various aspects in Section 4. Finally, we conclude the paper and give some comments for future work in Section 5.

## 2. Methodology

In this section, we first give a brief review of the guided normal filtering, and explain motivations of our mesh denoising method. Then, we introduce our two-stage scheme to construct adaptive consistent neighborhoods for computing a reliable guide normal field. Finally, we articulate the whole framework of our mesh denoising method.

### 2.1. Background of Guided Normal Filtering

Guided normal filtering [21] followed by vertex updating is a well developed feature-preserving mesh denoising framework. The key of guided normal filtering is that it provides a robust guidance normal for each face of the mesh. For each face, the guidance normal is obtained by averaging the face normals in a patch that contains the current face. Then, the joint bilateral filtering based on the computed guidance normal field is performed to get the filtered normal $n_{i}^{{}^{\prime }}$ of face $f_{i}$ as follows:

(1) $$n_{i}^{{}^{\prime }}=\frac{1}{\eta_{i}}\sum_{f_{j}\in P_{i}}A_{j}\rho_{r}\left(c_{i},c_{j}\right)\rho_{s}\left(g_{i},g_{j}\right)n_{j}\;,$$

where $\eta_{i}=∥\sum_{f_{j}\in P_{i}}A_{j}\rho_{r}\left(c_{i},c_{j}\right)\rho_{s}\left(g_{i},g_{j}\right)n_{j}{∥}_{2}$. $A_{j}$, $c_{j}$, $n_{j}$ are the area, centroid, and face normal of the face $f_{j}$ in the 1-ring neighborhood $P_{i}$ of the face $f_{i}$, respectively. $g_{i},g_{j}$ are the guidance normals of $f_{i},f_{j}$, respectively. $\rho_{r}\left(c_{i},c_{j}\right)=\exp\left(-\frac{∥c_{i}-c_{j}{∥}_{2}^{2}}{2\sigma_{r}^{2}}\right),\rho_{s}\left(g_{i},g_{j}\right)=\exp\left(-\frac{∥g_{i}-g_{j}{∥}_{2}^{2}}{2\sigma_{s}^{2}}\right)$ are the Gaussian functions, where $\sigma_{r},\sigma_{s}$ are variance parameters. $c_{i}$ is the centroid of the face $f_{i}$, and ${∥\cdot ∥}_{2}$ is the Euclidean norm. According to the Equation (1), the filtered face normals of the surface can be obtained, then we reconstruct vertex positions to match these filtered normals.

**Figure 1:**
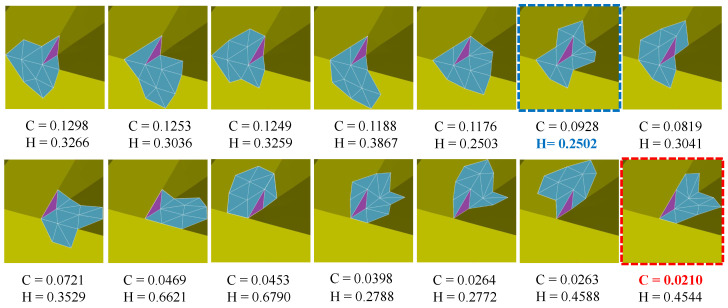
The consistent neighborhood selected by H [21] and C. C is computed from Equation (2). The neighborhood framed by red and blue dashed box are selected by C and H, respectively.

**Figure 2:**
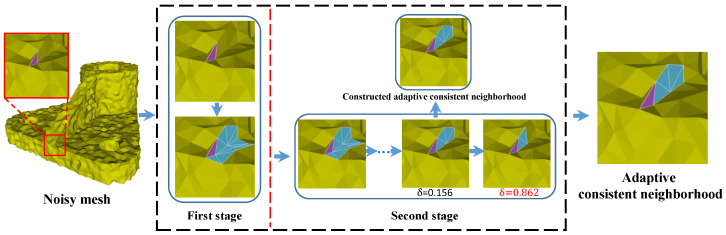
The pipeline of the proposed two-stage method for constructing adaptive consistent neighborhood. In the first stage, for a face (in purple), we compute a coarse consistent neighborhood by using patch-shift method with the proposed consistency measure. In the second stage, we propose a graph-cut based scheme for iteratively refining the obtained coarse consistent neighborhoods in a shape-aware manner.

Due to a robust estimation provided by the guidance for the true normals of the noisy mesh, guided normal filtering shows the superiority of sharp features preserving and robustness for noise. However, when the mesh contains complex shapes (e.g., narrow structures, multi-scale features, and fine details), they cannot get a proper guidance due to the unreasonable patch selected by the consistency measure $H(P_{ij})$. As we can see the Figure 1, according to the smallest value of the consistency measure $H(P_{ij})$, the most consistent neighborhood of the face (in purple) is selected, while the neighborhood contains sharp features more so that the guidance of the face (in purple) is not proper. Thus, the filtered results will blur sharp features in these regions. To obtain more faithful results in these regions, we propose a two-stage scheme to construct adaptive consistent neighborhoods for guided normal filtering. The construction pipeline is demonstrated in Figure 2. In the first stage, inspired by [21,39], we newly define a consistency measure to select a coarse consistent neighborhood for each face in a patch-shift manner. Then, a graph-cut based scheme is iteratively performed to adaptively construct different neighborhoods to match the corresponding local shapes of the mesh.

### 2.2. Coarse Consistent Neighborhood Selection

The consistent neighborhood is the key of recovering geometrical features and fine details in the denoised results from noisy mesh in the guided normal filtering (GNLF) framework. However, in the regions of narrow structures, multi-scale features, and fine details, a reliable consistent neighborhood can not be obtained in the GNLF framework so that the denoised results blur geometrical features and details. To solve this problem, we propose a two-stage method to obtain the adaptive consistent neighborhood for guided normal filtering. Our first-stage method aims to select a coarse consistent neighborhood for each face $f_{i}$ from all 1-ring neighborhoods that contain $f_{i}$. If all the faces in the neighborhood of $f_{i}$ have similar normal directions, then using this neighborhood to compute the guidance normal at $f_{i}$ will gets more faithful denoising results. However, it is hard to obtain this consistent neighborhood of $f_{i}$, especially in the regions of the complex shapes (e.g., narrow structures, multi-scale features, and fine details). As we can see the Figure 1, the consistent neighborhood that has the similar normal directions of the purple face can not be searched in all 1-ring neighborhoods that contain $f_{i}$ by using a consistency measure. For example, by using the consistency measure $H(P_{ij})$, the GNLF method gets worse neighborhoods in which the faces normals directions are disordered. Thus, to solve this problem, a new consistency measure is proposed to search a coarse consistent neighborhood for each face in a patch-shift manner, in which the normal directions of the faces are as similar as possible. In the second-stage, a graph-cut based scheme is iteratively performed in the coarse consistent neighborhood, which adaptively constructs different neighborhoods to match the corresponding local shapes of the mesh.

To evaluate the consistency of the neighborhood of $f_{i}$, the new consistency measure is as follows:

(2) $$C(P_{ij},f_{i})=F\left(P_{ij}\right)\cdot S\left(P_{ij},f_{i}\right),$$

where $P_{ij}$ is a 1-ring neighborhood of the face $f_{j}$ that contains the face $f_{i}$. $F(P_{ij})$ is used to measure the flatness of $P_{ij}$.

(3) $$F\left(P_{ij}\right)=\left(\max_{f_{j},f_{k}\in P_{ij}}{∥n_{j}-n_{k}∥}_{2}\right)\cdot \left(\frac{1}{{\bar{A}}_{P_{ij}}\left|P_{ij}\right|}\sum_{f_{j}\in P_{ij}}A_{j}{∥n_{j}-{\bar{n}}_{P_{ij}}∥}_{2}\right),$$

where $|P_{ij}|$ is the face number of the neighborhood $P_{ij}$, and ${\bar{A}}_{P_{ij}}=\frac{1}{|P_{ij}|}\sum_{f_{j}\in P_{ij}}A_{j}$ is the average area of all the faces in the neighborhood $P_{ij}$. ${\bar{n}}_{P_{ij}}=\frac{1}{∥\sum_{f_{j}\in P_{ij}}A_{j}n_{j}{∥}_{2}}\sum_{f_{j}\in P_{ij}}A_{j}n_{j}$ is the average value of the normals of each face in the neighborhood $P_{ij}$. The smaller of $F(P_{ij})$ means that the candidate neighborhood $P_{ij}$ is smoother.

$S(P_{ij},f_{i})$ is used to measure the similarity of the normal directions between the face $f_{i}$ and the neighborhoods $P_{ij}$, and the smaller value means more similar.

(4) $$S\left(P_{ij},f_{i}\right)=(\max_{f_{j}\in P_{ij}}∥n_{i}-n_{j}{∥}_{2})\cdot \left(∥n_{i}-{\bar{n}}_{P_{ij}}{∥}_{2}\right).$$

Thus, the product $C(P_{ij},f_{i})$ can measures the consistency of the neighborhood of the face $f_{i}$ well, and the coarse consistent neighborhood $P_{i}^{c}$ for $f_{i}$ in a patch-shift manner [21] can be searched by using $C(P_{ij},f_{i})$. As seen in Figure 1, a neighborhood of the face (in purple) is searched by using $C(P_{ij},f_{i})$, in which the normal directions of the faces are as similar as possible. Due to the narrow structures, the neighborhood also contains some geometric features. Thus, we perform a graph-cut based scheme in the neighborhood, which adaptively splits some faces in the different neighborhoods to match the corresponding local shapes of the mesh.

### 2.3. Adaptive Consistent Neighborhood Construction

When the mesh includes complex shapes (e.g., narrow structures, multi-scale features, and fine details), the coarse consistent neighborhood obtained by the first stage may still contain geometric features. In this case, if the coarse consistent neighborhood is used to calculate the guidance normal, which will blur sharp features. So, the second-stage strategy based on the graph cut scheme is proposed to iteratively split some faces from the first-stage consistent neighborhood, which can obtain an adaptive consistent neighborhood that has the more similar orientations with the current face. In each iteration, we firstly build a weighted graph based on the given neighborhood to obtain the indicator vector. Then, we use the indicator vector to bipartition the given neighborhood. Finally, a measure is introduced to judge the rationality of the segmented neighborhood for avoiding over-segmentation. The iterative graph cut scheme for obtaining the adaptive consistent neighborhood is sketched in Algorithm 1, and the main steps are as follows:

**(1)  Construct Laplacian matrix.** As graph construction has a crucial effect on the efficiency of the graph cut scheme, we firstly construct graphs over the given patch for our iterative graph cut scheme. We consider an undirected weighted graph $G_{p}=\left\{T,E,W\right\}$ composed of a node set $T=\{\tau_{i}:i=1,2,\ldots ,m\}$, an edge set *E* connecting nodes, and a similarity matrix *W*, where m is the number of the node set. *W* is a real symmetric m×m matrix, whose element $w_{i,j}$ in *i*-th row and *j*-th column is the weight assigned to the weighted edge connecting nodes $\tau_{i}$ and $\tau_{j}$. Our constructed graph $G_{p}$ is used to spilt some faces in the given patch based on the graph cut scheme, so *T* in the graph $G_{p}$ is the face set of the given patch.

In the graph cut scheme, the weight edge that is crossed the segmentation line of $G_{p}$ should have a relatively low-value. To this aim, if the faces correspond to $\tau_{i}$ and $\tau_{j}$ which not share a common edge, we set $w_{i,j}$ equal to 0; otherwise, $w_{i,j}$ is set as follows:

(5) $$w_{i,j}=\alpha \cdot \exp\left(-\frac{∥n_{\tau_{i}}-n_{\tau_{j}}{∥}_{2}^{2}}{\sigma_{p}^{2}}\right),$$

where $n_{\tau_{i}}$, $n_{\tau_{j}}$ are the face normals of the given patch that correspond to nodes $\tau_{i}$ and $\tau_{j}$. $\sigma_{p}$ is a scaling parameter, which controls the decreasing speed of $w_{ij}$. Empirically, we set $\sigma_{p}$ equal to 0.8 in our experiments. α is defined as:

(6) $$\alpha =\left\{\begin{array}{c} 0.1,\;∠(n_{\tau_{i}},n_{\tau_{j}})\geq \theta \\ \;\;\;1,\;∠(n_{\tau_{i}},n_{\tau_{j}})<\theta \end{array}\right.,$$

where θ is a user-specified angle threshold for identifying the geometry feature, which will be discussed in Section 4. Then, we build the corresponding Laplacian matrix $L\in R^{m\times m}$ of the graph $G_{p}$, which can be written as:

(7) L=D−W,

where $D=diag(D_{1},...,D_{m})$, and $D_{i}=\sum_{i=0}^{m}w_{ij}$ is the sum of the *i*-th row elements in the similarity matrix *W*.

**(2) Obtain segmented result.** According to the generalized eigensystem [40] of the Laplacian matrix L, the eigenvector $\chi =\{\chi_{1},\chi_{2},...,\chi_{m}\}$ corresponding to the second smallest eigenvalue of the eigensystem is obtained. Each element in the eigenvector χ corresponds to a node in the graph $G_{p}$, and the value of the elements represents the geometric distribution of the nodes in the graph $G_{p}$. We sort the elements of the vector χ in increasing order, and then we get the sorted vector $\hat{\chi }$. φ(·) is a index mapping from the vector χ to the sorted vector $\hat{\chi }$, e.g., $\chi_{i}={\hat{\chi }}_{\varphi (i)}$ and $\chi_{\varphi^{-1}\left(i\right)}={\hat{\chi }}_{i}$. The jump point in the sorted vector $\hat{\chi }$ means there is a splitting point in the graph $G_{p}$. In order to find the jump point effectively, we first build the first-order difference vector $\nabla \hat{\chi }=\left\{{\hat{\chi }}_{2}-{\hat{\chi }}_{1}\;,...,\;{\hat{\chi }}_{\left|p\right|}-{\hat{\chi }}_{m-1}\right\}\in R^{m-1}$. Then, we search the largest value of the vector $\nabla \hat{\chi }$ and record the corresponding index in the $\nabla \hat{\chi }$ as λ. The index mapping φ(·) can be used to obtain the original order in the eigenvector χ, and we can use the splitting index $\phi^{-1}\left(\lambda \right)$ to divide the nodes in graph $G_{p}$ into two sets:

(8) $$A=\left\{\tau_{\phi^{-1}\left(1\right)}\;,...,\;\tau_{\phi^{-1}\left(\lambda \right)}\right\}\;\&\;B=\left\{\tau_{\phi^{-1}\left(\lambda +1\right)}\;,...,\;\tau_{\phi^{-1}\left(m\right)}\right\}.$$

Finally, the set containing the current face is selected as the intermediate result of the adaptive consistent neighborhood.

**(3) Compute stopping criteria**. To avoid over-segmentation, a measure $\delta (P_{k},P_{k+1},f_{i})$ is proposed as follows:

(9) $$\delta \left(P_{i}^{k},P_{i}^{k+1},f_{i}\right)=\frac{C(P_{i}^{k+1},f_{i})}{C(P_{i}^{k},f_{i})+\varepsilon }\;\;\;,$$

where ε is a small positive number to avoid zero division, and $P_{i}^{k},P_{i}^{k+1}$ are the neighborhood of the face $f_{i}$ of the last iteration and the segmented neighborhood of the face $f_{i}$, respectively. C(·,·) is our proposed consistency measure, which is defined in (2). $\delta (P_{i}^{k},P_{i}^{k+1},f_{i})$ measures the consistency change between $P_{i}^{k}$ and $P_{i}^{k+1}$. A threshold β is introduced to manually tune the segmented degree through (9). If $\delta (P_{i}^{k},P_{i}^{k+1},f_{i})<\beta$, our iterative graph cut scheme continues with the previous segmented result as the input patch; otherwise, the iterative scheme is stopped and the final segmented neighborhood $P_{i}^{*}=P_{i}^{k}$ is outputted.
**Algorithm 1:** Adaptive consistent neighborhood construction.
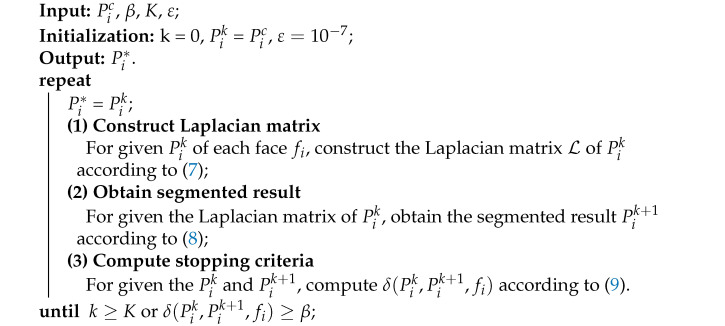



### 2.4. Guided Normal Filtering with Adaptive Consistent Neighborhood

Through our two-stage scheme, the adaptive consistent neighborhoods that contain geometric features as few as possible are constructed to provide a robust estimation of the guidance normals for the noisy mesh. Then, the filtered face normals are obtained by the joint bilateral filtering with the guidance. Finally, the vertex positions are reconstructed to match the filtered face normals. The whole iterative framework is listed in Algorithm 2, and the main steps are as follows. The corresponding pipeline of our method can be seen in Figure 2.

(1) **Compute face normals of the input mesh**. A mesh of arbitrary topology without any degenerate triangle is repesented as $M\in R^{3}$, and the corresponding faces set and vertices set are denoted as $\left\{f_{i}:i=1,2,\ldots ,F\right\}$ and $\left\{v_{i}:i=1,2,\ldots ,V\right\}$, respectively. Here, F and V are the number of faces and vertices in the *M*, respectively. For a face $f_{i}$, its outward unit normal can be calculated as:

(10) $$n_{i}=R\left(\left(v_{i_{2}}-v_{i_{1}}\right)\times \left(v_{i_{3}}-v_{i_{1}}\right)\right),$$

where $R\left(\cdot \right)=\frac{\cdot }{{∥\cdot ∥}_{2}}$, and $v_{i_{1}},v_{i_{2}},v_{i_{3}}$ are the positions of the three vertices of the $f_{i}$ in a fixed orientation, respectively.

(2) **Compute guidance normals**. Through our two-stage scheme, the adaptive consistent neighborhood is obtained for each face $f_{i}$ of the input noisy mesh. Then, we can use the neighborhoods to compute the guidance normal $g_{i}$ at each face $f_{i}$.

(11) $$g_{i}=R\left(\sum_{f_{j}\in P_{i}^{*}}A_{j}n_{j}\right),$$

where $A_{j},n_{j}$ are the area, the face normal of the face $f_{j}$ in the neighborhood $P_{i}^{*}$. $P_{i}^{*}$ is the adaptive consistent neighborhood of the face $f_{i}$.

(3) **Compute filtered normals**. The robust estimation of the guidance for the true normals of the noisy mesh is obtained by our adaptive consistent neighborhood, we can use Equation (1) to compute filtered normals.

(4) **Reconstruct vertex positions of the mesh**.  After obtaining the filtered face normal field, we should update vertex positions of the mesh to match the filtered face normal field. To this end, we use a classical vertex updating scheme presented by Sun et al. [14]. Specifically, we reposition vertex positions V by solving the following minimization problem:

(12) $$\min_{V}\left\{\sum_{f_{k}}\sum_{\left(v_{i},v_{j}\right)\in f_{k}}{\left(n_{k}^{{}^{\prime }}\cdot \left(v_{i}-v_{j}\right)\right)}^{2}\right\},$$

where $n_{k}^{{}^{\prime }}$ is the filtered face normal of $f_{k}$. By using gradient descent to solve the problem (12), we reconstruct vertex positions by the following iterative formula:

(13) $$v_{i}^{{}^{\prime }}=v_{i}+\frac{1}{\left|\Gamma (i)\right|}\sum_{f_{t}\in \Gamma \left(i\right)}n_{t}^{{}^{\prime }}\left(n_{t}^{{}^{\prime }}\cdot \left(c_{t}-v_{i}\right)\right),$$

where $v_{i}^{{}^{\prime }}$ is the updated vertex of $v_{i}$. Γ(i) is the set of mesh faces that share a common mesh vertex $v_{i}$, and |Γ(i)| is the number of faces contained in Γ(i). $c_{t}$ is the centroid of $f_{t}$. More details can refer to the work [14].
**Algorithm 2:** Our mesh normal filtering framework.
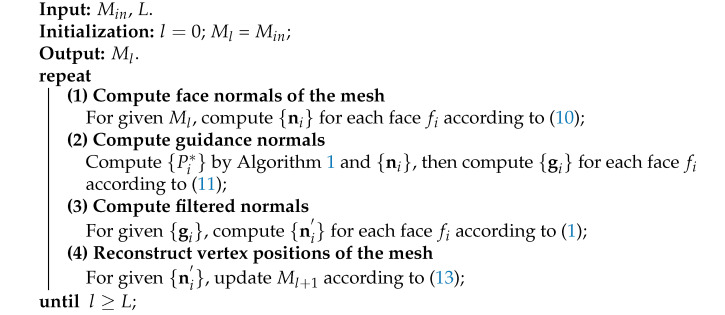



## 3. Experiment Results

The new method is implemented with C++ language. CGAL, Eigen and OpenGL library are used to develop the software on Microsoft Visual Studio 2010. A group of CAD, non-CAD and original models scanned by 3D scanning equipments are used to conduct a set of experiments. All of the experiments are run on the same server with an Intel i7 1.8 GHz CPU and 16GB RAM. In order to analyze the performance of our new method, five typical methods are chosen to conduct a group of comparisons, such as BNLF [15], L0M [22], ROFI [17], CNLR [35], and GNLF [21].

For all our experiments, ε,β,K,L are the same. Specifically, ε is a small positive number to avoid zero division, which is empirically fixed as $10^{-7}$. β is a threshold for tuning the segmented degree, which is empirically set as 0.6. K=3 and L=10 are the maximum iteration numbers of segmentation and the face normal filtering method, respectively.

### 3.1. Qualitative Comparisons

**Figure 3:**
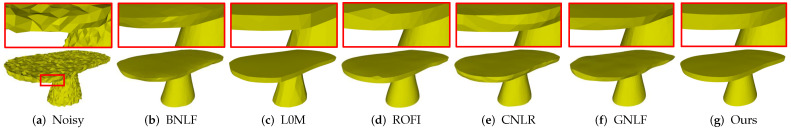
Denoising results of Table model.

Firstly, we conduct a group of experiments by using a Table model, which includes narrow and smooth geometrical structures. As shown in Figure 3, Figure 3a is the noisy mesh with 0.2 Gaussian noise. It obviously indicates that these five methods both perform well for eliminating the noise. However, most of these methods blur sharp features in varying degrees, except methods L0M and ours. Specifically, both methods BNLF and ROFI cannot preserve sharp features, due to that these two methods are based on the bilateral filter, which cannot distinguish sharp features from noise clearly; see Figure 3b,d. Since its performance depends on the completeness of the training dataset, the learning-based method CNLR blurs sharp features; see Figure 3e. Because it is short of the robustness to mesh topology, method GNLF is failed to keep narrow structures; see Figure 3f. Although method L0M can recover sharp features well, it produces staircase effects in smoothly curved regions due to its high requirements of sparsity; see Figure 3c. On the contrary, our method can preserve sharp features on narrow structures and recover smoothly curved regions well; see Figure 3g.

**Figure 4:**
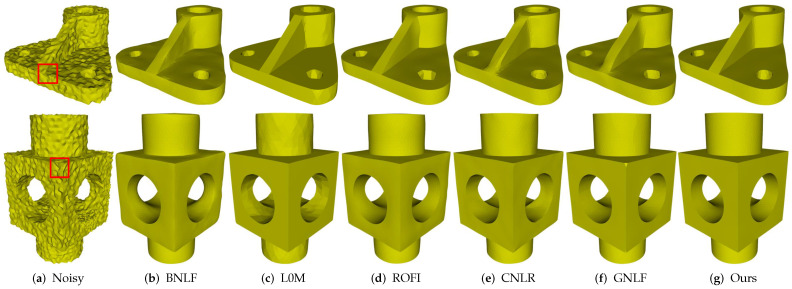
Denoising results of Part and Block models.

Figure 4 demonstrates the denoising results of Part and Block, which contain sharp features on highly irregular sampled regions. The two models shown in Figure 4a are the noisy meshes with 0.2 Gaussian noise. Although both methods BNLF and CNLR can recover smooth features well, they inevitably blur sharp features; see Figure 4b,e. On the contrary, although methods L0M and ROFI can preserve sharp features, they cannot recover smoothly curved regions well. Specifically, both two methods usually produce staircase effects in these regions; see Figure 4c,d. Method GNLF can recover smooth regions while keeping most of sharp features well. However, it sometimes blurs corners because it often lacks robustness to mesh topology nearing corner features; see Figure 4f. Compared to these state-of-the-arts, our method can recover sharp features on highly irregular sampled regions; see Figure 4g.

**Figure 5:**
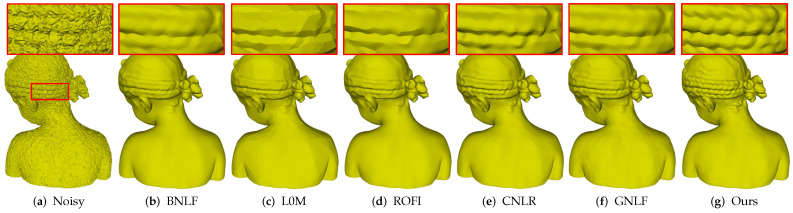
Denoising results of Girl model.

Figure 5 demonstrates denoising results of Girl, which is a non-CAD mesh with multi-scale features. As we can see, Figure 5a is the noisy mesh with 0.2 Gaussian noise. Method BNLF over-smooths small-scale features in the zoomed-in view areas; see Figure 5b. Methods L0M and ROFI flatten those small-scale features while producing false edges on smooth regions; see Figure 5c,d. Methods CNLR and GNLF can recover large-scale features, but they also blur those small-scale features; see Figure 5e,f. Compared to these methods, our method can remove noise effectively while keeping multi-scale features; see Figure 5g.

**Figure 6:**
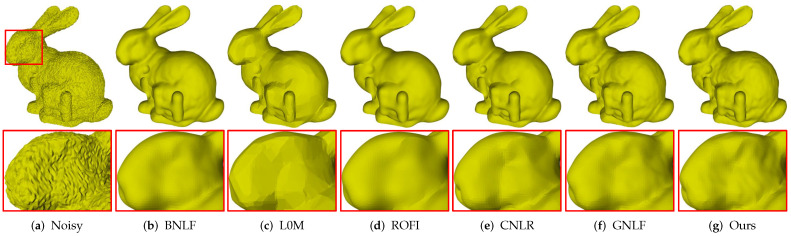
Denoising results of Bunny model.

Figure 6 gives comparisons on Bunny containing fine details. As can be seen, Figure 6a is the noisy mesh with 0.2 Gaussian noise. Methods BNLF, CNLR, and GNLF over-smooth geometric details in varying degrees; see Figure 6b,e,f. Both methods L0M and ROFI suffer the over-sharpened effects. Besides, method L0M also produces false edges in smooth regions while method ROFI blurs the small-scale features; see Figure 6c,d. Compared to the above methods, our method produces the visually best result with the most details preserved; see Figure 6g.

**Figure 7:**
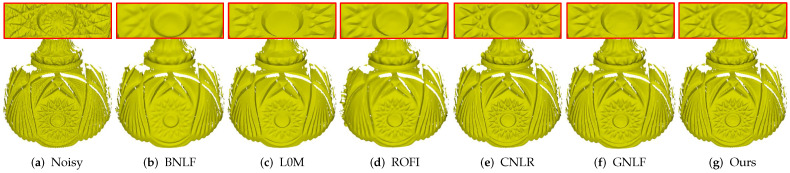
Denoising results of a laser-scanned mesh.

Figure 7 demonstrates the comparison on a laser-scanned mesh with rich geometric details. As can be seen, all the methods can remove noise effectively. Moreover, method BNLF over-smooths the details; see Figure 7b. Although methods L0M, ROFI, CNLR, and GNLF can recover those relatively large-scale geometric details, all of them blur the small-scale details; see in Figure 7c–f. Specifically, methods L0M, ROFI, and GNLF also cause over-sharpened effects. Compared to the above state-of-the-art methods, our method can not only keep those relatively large-scale details well, but also preserves the most small-scale details; see Figure 7g. These results demonstrate that our method outperforms the compared methods in handling laser-scanned meshes.

**Figure 8:**
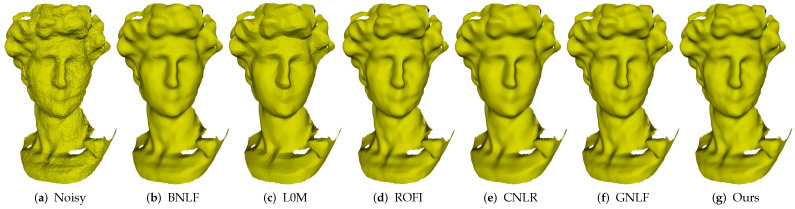
Denoising results of David scanned by Kinect.

**Figure 9:**
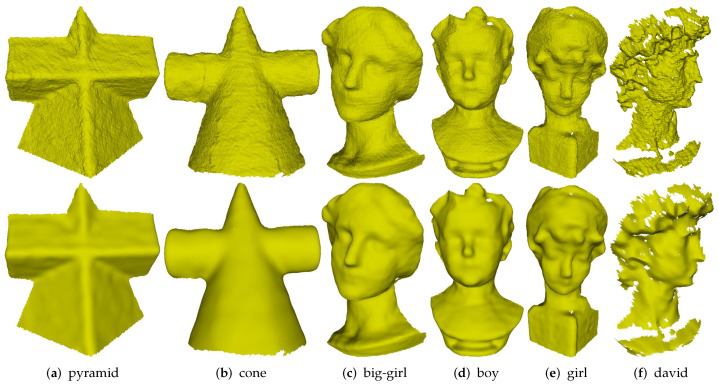
Denoising results of Kinect-scanned models.

Recently, a lot of triangulated meshes are obtained using consumer-grade depth cameras, e.g., Microsoft Kinect. In Figure 8, we show the denoising results of David, which is scanned by Kinect. As can be seen, all the methods can effectively remove noise while preserving the geometric features well. Similarly, method L0M produces many false edges in smoothly curved regions for its highest sparsity requirements; see Figure 8c. However, according to the quantitative comparison results (MSAE) in the next subsection, we can see that the errors of our method are the lowest when compared to the above state-of-the-art methods. Thus, our method outperforms other compared methods in handling meshes acquired by Kinect. In addition, we give more denoising results on the raw data scanned by Kinect in Figure 9. As we can see, our method can consistently produce satisfactory results.

### 3.2. Quantitative Comparisons

**Figure 10:**
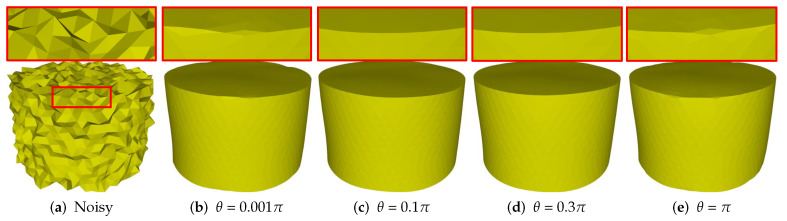
Denoising results for different θ (0.001π, 0.1π, 0.3π, π).

**Figure 11:**
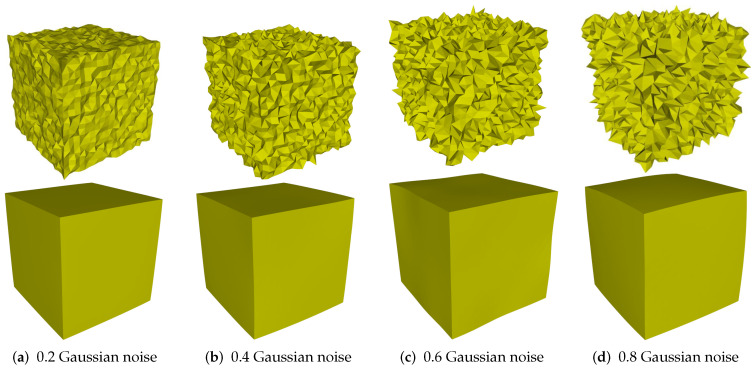
Denoising results of Cube model.

**Figure 12:**
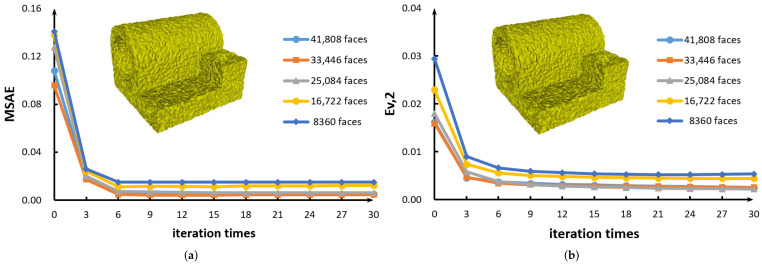
(**a**) Error curves of MSAE for Joint model with five different resolutions. (**b**) Error curves of $E_{v,2}$ for Joint model with five different resolutions.

In Section 3.1, the effectiveness of our new method has been verified by rendering these 3D models, see Figure 3, Figure 4, Figure 5, Figure 6, Figure 7, Figure 8 and Figure 9. In this section, MSAE and $E_{v,2}$ are exploited to analyze the deviation between the denoised meshes and groundtruth [15,21], see Table 1, Figure 10 and Figure 11. Computing time is also collected to analyze the computational intensity [41,42,43] of different methods, see Figure 12.

As shown in Table 1 and Figure 10, the MSAE of our method is the minimum one, which means that the deviation between the face normals of denoised meshes and ground-truth is minimal. It indicates that the adaptive consistent neighborhood exploited by our method can effectively generate more accurate face normal field. In addition, the $E_{v,2}$ of our method is lower than most of the others, see Figure 11. The $E_{v,2}$ of ROFI is bigger than ours, but sharp edges may be generated in some small-scale smooth regions, see Figure 8d.

The complexity of an algorithm directly impacts the computing time of the method. The new method is developed based on GNLF. In our method, adaptive consistent neighborhood is exploited to compute guided face normal field. This procedure is computational intensive. It increases the complexity of the method, so the computing time of our method is longer than GNLF. However, the computing time of our method is not the longest one when compared with other methods, see Figure 12.

## 4. Discussion

### 4.1. Parameter Setting

Similar to existing methods, our method needs to tune several parameters to produce satisfactory results. The parameter θ is used to recognize sharp features from noise. Figure 10 shows the results of different θ with fixed other parameters. As can be seen that, Figure 10a is the noisy mesh with 0.35 Gaussian noise. When the value of θ is too small or too large, our method can blur sharp features; see Figure 10b,e. The reason is that our method can recognize all the edges as features or non-features if the value of θ is too small or too large. Besides, there is a range of θ values can be used to generate satisfactory results with sharp features well preserved; see Figure 10c,d. In our experiments, we empirically set θ in the range of [0.1π,0.3π].

### 4.2. Robustness Test

In this subsection, we discuss the robustness of our method against different levels of noise. As can be seen in Figure 11, Figure 11a–d are the noisy meshes with 0.2, 0.4, 0.6, and 0.8 Gaussian noise, respectively. When the mesh corrupted by small-scale noise (standard deviation less than 0.4 mean edge length), our method can effectively remove noise while preserving sharp features well; see Figure 11a,b. However, when the level of noise increases, our method cannot preserve the original shape of the surface; see Figure 11c,d.

We also perform two robustness tests on MSAE and $E_{v,2}$ for different mesh resolutions in Figure 12. As can be seen, when the mesh resolution keeps decreasing, the values of MSAE and $E_{v,2}$ do not change significantly. Therefore, our method is robust and reliable.

## 5. Conclusions

The main novelty and findings of this paper are as follows. Firstly, we design a new consistency measurement to explicitly select the coarse consistent neighborhood containing the fewest geometric features. Then, we further present an improved technique based on graph-cut to adaptively construct the more accurate neighborhood that does not contain any geometric features. By using the constructed consistent neighborhoods, we can calculate an accurate guided normal field. The adaptive consistent neighborhood of each face is built by the proposed two-stage approach, which is the key part of this paper. By constructing the adaptive consistent neighborhood of each face, we can neglect the influence from features in the local neighborhood of the face. Based on the constructed adaptive consistent neighborhoods, we apply the guided normal filtering method to restore the noisy normal vector field. Then, vertex positions are reconstructed to match the filtered normal vector field. Our mesh denoising method preserves geometric features well, and is robust against complex topologies (e.g., narrow structures). We have compared our mesh denoising method with the state-of-the-art methods visually and numerically, and discussed our methods from various aspects.

Although our mesh denoising method performs better than the compared state-of-the-art methods, the CPU cost of our method is intensive. Because our graph cut algorithm is performed sequentially for each mesh face, it can potentially be parallelized by using OpenMP or CUDA in future work. Moreover, we plan to extend our consistency measurement and adaptive guided normal filtering method to point clouds.

## Figures and Tables

**Table 1:** Quantitative evaluation results of the tested mesh denoising methods.

Mesh	$\mathrm{MSAE}(\times 10^{-3})$, $E_{v,2}\left(\times 10^{-3}\right)$; Time (in Seconds)											
	BNLF	L0M	ROFI	CNLR	GNLF	Ours
Table	18.3, 2.45; 0.08	7.39, 2.14; 1.50	6.53, 4.21; 3.61	12.9, 2.02; 16.7	31.2, 3.49; 0.55	**5.88**, **1.03**; 2.43
Part	6.54, 3.63; 0.06	9.38, 4.57; 1.66	10.7, 2.89; 3.14	5.73, 2.88; 16.8	8.15, 2.91; 0.64	**4.08**, **2.43**; 2.06
Block	12.3, 3.35; 0.12	5.60, 2.36; 4.27	3.89, 3.27; 6.96	2.53, 2.42; 17.4	5.06, 1.91; 1.03	**2.37**, **1.78**; 4.33
Child	37.1, 1.22; 0.57	49.7, 1.50; 34.3	47.1, 1.20; 53.8	31.1, 1.14; 23.1	42.7, 1.15; 10.1	**19.8**, **0.94**; 22.6
Bunny	50.4, 5.12; 0.35	31.5, 2.70; 23.1	27.2, 2.02; 33.1	13.7, 1.24; 20.9	48.3, 5.18; 8.72	**12.6**, **1.15**; 14.7
David	99.6, 7.34; 0.52	95.7, 6.95; 23.2	85.6, **5.86**; 39.2	99.3, 6.25; 21.0	90.6, 6.46; 14.5	**82.6**, 6.32; 30.9

## Data Availability

The experimental datasets are available at https://pan.baidu.com/s/1zgbTq2SGDDSaPwM4VF23OQ (download code:ieec).

## Abbreviations

The following abbreviations are used in this manuscript:

BNLF	bilateral normal filtering
L0M	L0 minimization
ROFI	robust and high fidelity mesh denoising
CNLR	cascaded normal regression
GNLF	guided normal filtering
MSAE	mean square angular error
$E_{v,2}$	$L_{2}$ vertex-based error

## References

[1] Wang J., Zhang X., Yu Z. (2012). A cascaded approach for feature-preserving surface mesh denoising. Comput. Aided Des..

[2] Liu Z., Xiao X., Zhong S., Wang W., Li Y., Zhang L., Xie Z. (2020). A feature-preserving framework for point cloud denoising. Comput. Aided Des..

[3] Liu Z., Zhong S., Xie Z., Wang W. (2019). A novel anisotropic second order regularization for mesh denoising. Comput. Aided Geom. Des..

[4] He Z., Deng M., Xie Z., Wu L., Chen Z., Pei T. (2020). Discovering the joint influence of urban facilities on crime occurrence using spatial co-location pattern mining. Cities.

[5] Liu Z., Wang W., Zhong S., Zeng B., Liu J., Wang W. (2020). Mesh denoising via a novel Mumford-Shah framework. Comput. Aided Des..

[6] Taubin G. A signal processing approach to fair surface design. Proceedings of the 22nd Annual Conference on Computer Graphics and Interactive Techniques.

[7] Desbrun M., Meyer M., Schroder P., Barr A.H. Implicit fairing of irregular meshes using diffusion and curvature flow. Proceedings of the 26th Annual Conference on Computer Graphics and Interactive Techniques.

[8] Desbrun M., Meyer M., Schröder P., Barr A.H. (2000). Anisotropic feature-preserving denoising of height fields and bivariate data. Graph. Interface.

[9] Yagou H., Ohtake Y., Belyaev A.G. Mesh smoothing via mean and median filtering applied to face normals. Proceedings of the Geometric Modeling and Processing. Theory and Applications.

[10] Bajaj C.L., Xu G. (2003). Anisotropic diffusion of surfaces and functions on surfaces. ACM Trans. Graph..

[11] Jones T.R., Durand F., Desbrun M. (2003). Non-iterative, feature-preserving mesh smoothing. ACM Trans. Graph..

[12] Fleishman S., Drori I., Cohen-Or D. (2003). Bilateral mesh denoising. ACM Trans. Graph..

[13] Taubin G. (2001). Linear Anisotropic Mesh Filters.

[14] Sun X., Rosin P.L., Martin R.R., Langbein F.C. (2007). Fast and effective feature-preserving mesh denoising. IEEE Trans. Vis. Comput. Graph..

[15] Zheng Y., Fu H., Au O.K.C., Tai C.L. (2011). Bilateral normal filtering for mesh denoising. IEEE Trans. Vis. Comput. Graph..

[16] Lu X., Deng Z., Chen W. (2015). A robust scheme for feature-preserving mesh denoising. IEEE Trans. Vis. Comput. Graph..

[17] Yadav S.K., Reitebuch U., Polthier K. (2018). Robust and high fidelity mesh denoising. IEEE Trans. Vis. Comput. Graph..

[18] Zhang J., Deng B., Hong Y., Peng Y., Qin W., Liu L. (2018). Static/dynamic filtering for mesh geometry. IEEE Trans. Vis. Comput. Graph..

[19] Xing Y., He Y., He L., Zha W., Tan J. (2020). A dynamic and adaptive scheme for feature-preserving mesh denoising. Graph. Model..

[20] Pan W., Lu X., Gong Y., Tang W., Liu J., He Y., Qiu G. (2020). HLO: Half-kernel Laplacian operator for surface smoothing. Comput. Aided Des..

[21] Zhang W., Deng B., Zhang J., Bouaziz S., Liu L. (2015). Guided mesh normal filtering. Comput. Graph. Forum.

[22] He L., Schaefer S. (2013). Mesh denoising via *ℓ*_0_ minimization. ACM Trans. Graph..

[23] Zhang H., Wu C., Zhang J., Deng J. (2015). Variational mesh denoising using total variation and piecewise constant function space. IEEE Trans. Vis. Comput. Graph..

[24] Wu X., Zheng J., Cai Y., Fu C.W. (2015). Mesh denoising using extended ROF model with *ℓ*_1_ fidelity. Comput. Graph. Forum.

[25] Liu Z., Lai R., Zhang H., Wu C. (2019). Triangulated surface denoising using high order regularization with dynamic weights. SIAM J. Sci. Comput..

[26] Zhong S., Xie Z., Wang W., Liu Z., Liu L. (2018). Mesh denoising via total variation and weighted laplacian regularizations. Comput. Animat. Virtual Worlds.

[27] Lu X., Chen W., Schaefer S. (2017). Robust mesh denoising via vertex pre-filtering and l1-median normal filtering. Comput. Aided Geom. Des..

[28] Guo M., Han C., Wang W., Zhong S., Lv R., Liu Z. (2020). A novel truncated nonconvex nonsmooth variational method for SAR image despeckling. Remote Sens. Lett..

[29] Zhao Y., Qin H., Zeng X., Xu J., Dong J. (2018). Robust and effective mesh denoising using L0 sparse regularization. Comput. Aided Des..

[30] Zhong S., Xie Z., Liu J., Liu Z. (2019). Robust mesh denoising via triple sparsity. Sensors.

[31] Li X., Zhu L., Fu C.W., Heng P.A. (2018). Non-local low-rank normal filtering for mesh denoising. Comput. Graph. Forum.

[32] Wei M., Huang J., Xie X., Liu L., Qin J. (2019). Mesh denoising guided by patch normal co-filtering via kernel low-rank recovery. IEEE Trans. Vis. Comput. Graph..

[33] Chen H., Huang J., Remil O., Xie H., Qin J., Guo Y., Wei M., Wang J. (2019). Structure-guided shape-preserving mesh texture smoothing via joint low-rank matrix recovery. Comput. Aided Des..

[34] Lu X., Schaefer S., Luo J., Ma L., He Y. (2020). Low rank matrix approximation for 3D geometry filtering. IEEE Trans. Vis. Comput. Graph..

[35] Wang P.S., Liu Y., Tong X. (2016). Mesh denoising via cascaded normal regression. ACM Trans. Graph..

[36] Wang J., Huang J., Wang F.L., Wei M., Xie H., Qin J. (2019). Data-driven geometry-recovering mesh denoising. Comput. Aided Des..

[37] Bei W., Guo M., Huang Y. (2019). A Spatial Adaptive Algorithm Framework for Building Pattern Recognition Using Graph Convolutional Networks. Sensors.

[38] Guo M., Liu H., Xu Y., Huang Y. (2020). Building Extraction Based on U-Net with an Attention Block and Multiple Losses. Remote Sens..

[39] Li T., Wang J., Liu H., Liu L.g. (2017). Efficient mesh denoising via robust normal filtering and alternate vertex updating. Front. Inf. Technol. Electron. Eng..

[40] Shi J., Malik J. (2000). Normalized cuts and image segmentation. IEEE Trans. Pattern Anal. Mach. Intell..

[41] Guo M., Han C., Guan Q., Huang Y., Xie Z. (2020). A universal parallel scheduling approach to polyline and polygon vector data buffer analysis on conventional GIS platforms. Trans. GIS.

[42] Guo M., Guan Q., Xie Z., Wu L., Luo X., Huang Y. (2015). A spatially adaptive decomposition approach for parallel vector data visualization of polylines and polygons. Int. J. Geogr. Inf. Sci..

[43] Guo M., Huang Y., Guan Q., Xie Z., Wu L. (2017). An efficient data organization and scheduling strategy for accelerating large vector data rendering. Trans. GIS.

